# Concept for a Fan-beam Computed Tomography Image-guided Radiotherapy Device

**DOI:** 10.7759/cureus.4882

**Published:** 2019-06-11

**Authors:** Vincent Weidlich, Lawrence Lechuga, Dennis Dore, Georg A. Weidlich, Bill Nighan

**Affiliations:** 1 Medical Physics, Kingston University, Kingston upon Thames, GBR; 2 Medical Physics, University of Wisconsin Madison, Madison, USA; 3 Miscellaneous, ETM Electromatic Inc., Newark, USA; 4 Radiation Oncology, National Medical Physics and Dosimetry Company, Palo Alto, USA

**Keywords:** radiotherapy, fan beam igrt, fan beam ct simulation

## Abstract

Many advancements taking place in the field of radiation therapy come in the form of increasingly powerful devices and specialized treatments that aim to increase precision, visualization, and facility throughput. Although these devices are very effective at their respective roles within radiotherapy, they are expensive and require specialized vaults to shield the public and the radiation worker from the ionizing radiation. A proposed device, known as the Simple XRT, is designed to circumvent the inherent drawbacks of the current devices. The Simple XRT uses a 6 MV linear accelerator that utilizes diagnostic quality computed tomography (CT) image guidance. Simple XRT will serve as a cost-effective device for treating most cancer indications.

## Introduction

In the field of therapeutic radiation, many advances are continually taking place. From the early days of minimally image-guided radiation therapy, the field has evolved into precision stereotactic radiosurgery (SRS) and stereotactic body radiotherapy (SBRT) [1] and to optimizations in treatment time, dose rate, dose uniformity, precision, and image guidance. Examples of new developments in radiation therapy are Varian’s unflattened beam (Varian Medical Systems, Palo Alto, CA) [2], Tomotherapy’s large field dose painting [3], the robotic flexibility of the Accuray CyberKnife (Accuray Inc., Sunnyvale, CA) [4], Varian’s Unique system [5-6], VERO system (BrainLAB AG, Feldkirchen, Germany) [7-9], Versa HD (Elekta AB, Stockholm, Sweden) [10], Meridian (ViewRay, Sunnyvale, CA) [11], the RefleXion system (RefleXion Medical Inc., Hayward, CA) with biologic guidance [12], and most relevant, the Zap-X system (ZAP Surgical Systems Inc., San Carlos, CA) [13]. The focus of the field seems to be on highly specialized treatments that increase precision, visualization, and throughput of a facility. Although these cutting-edge systems are continually creating new possibilities for treatments that were not previously available, they also present a robust price tag, require highly trained personnel, and specially equipped vaults for treatment. The gyroscopically constructed Zap-X system is the exception while being clearly innovative and focused on intra-cranial and Head and Neck treatments of extreme accuracy at a low cost. The system proposed here was designed to treat all major radiotherapy indications except stereotactic radiosurgery.

There are numerous systems that are available and being developed in today’s market. From companies like Varian Medical Systems, Accuray, Elekta, Brainlab, RefleXion, and ViewRay a wide variety of treatments are available from very broad and versatile, to highly specialized and exotic. These systems can deliver radiation via X-rays or gamma rays and utilize specialized treatment procedures like intensity modulated radiation therapy (IMRT), tomotherapy, brachytherapy, or volumetric modulated arc therapy (VMAT). However, the more complex and powerful systems tend to increase the system cost, the shielding requirements for the vault in which it will be installed, and the level of specialization of the staff at the facility.

Nearly all of these devices are required to be installed in an appropriately equipped vault which adds significant cost to the project due to required construction. The resulting combined expense of the device, facility, and personnel requires a very large financial investment. This hurdle presents an opportunity to introduce a new low-cost device to the global market that can minimize the investment and infrastructure required to utilize quality radiation therapy resources.

The device proposed here, the Simple XRT, can be utilized to address some of the common drawbacks associated with the current devices on the market. The device will utilize a 6 MV linear accelerator and diagnostic quality computed tomography (CT) imaging to generate CT simulation imaging as well as daily pre-treatment image guidance. Furthermore, the system will require less shielding than conventional linear accelerators, which will reduce the required infrastructure to house this system. This system will be cost-effective and will be capable of treating most indications of cancer. Implementing this device will increase access to radiotherapy resources, which will reduce the overall economic and personal financial burden that cancer carries along with it.

## Technical report

**Figure 1:**
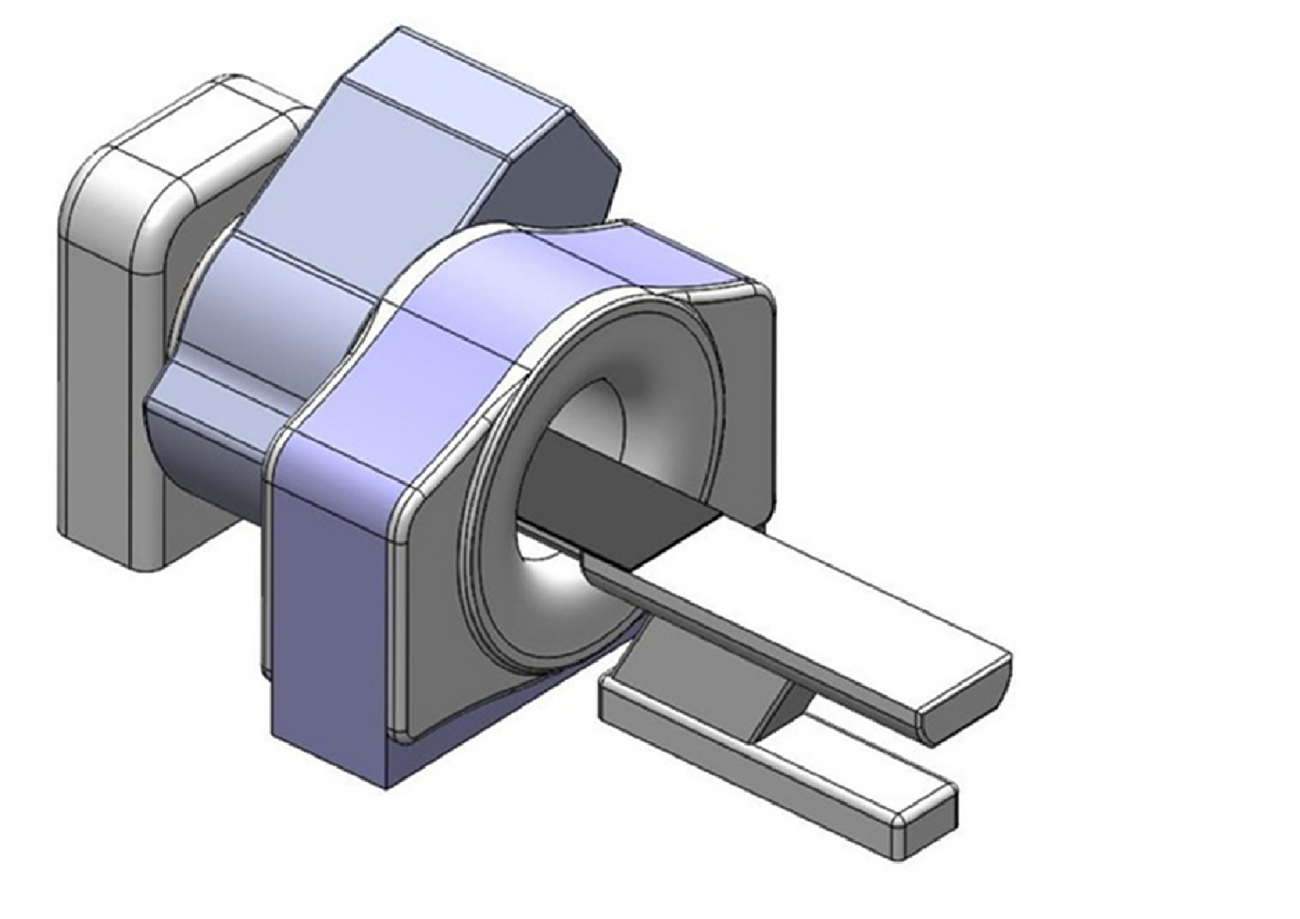
The proposed design for the Simple-XRT system Image courtesy ETM Electromatic Inc., Newark, CA.

The novel device shown in Figure 1 is called the Simple XRT and can be utilized in most hospitals or treatment facilities. Additionally, the proposed system utilizes the diagnostic quality CT technology, which presents significant advantages compared to alternative X-ray imaging modalities.

The Simple XRT system is mounted adjacent to a CT scanner that will function as CT simulator as well as for daily image guidance. The patient table of the CT scanner will be shared and utilizes a table extension that will allow patient positioning in the center of the treatment device after the completion of the imaging process effectively extending the longitudinal range of couch travel. Improvements for table rigidity ensure a deflection of no more than 3 mm at its full extent which was deemed acceptable. The treatment will be delivered in a single or multiple pass VMAT employing the use of a multi-leaf collimator (MLC) for modulation.

System imaging

The geometry of the employed fan-beam CT system (FBCT) prevents nearly all in-patient scattering from reaching the detector. Only the primary radiation that has passed through and has been attenuated by the patient will reach the detector and become part of the reconstructed data. Many thin slices are acquired as the kV X-ray tube rotates around the patient in seconds. The images acquired via fan beam technology have superior spatial and contrast resolution, compared to cone beam CT (CBCT) which is dominated by large amounts of scatter radiation that occurs within the large treatment beam. Utilizing FBCT for day-of-treatment imaging has the potential for less error when matching the diagnostic images to the day of treatment images. In turn, the improved set up and more precise images provide a more accurate treatment. The initial setup is performed on the CT scanner.

The Simple XRT system will allow a hospital’s existing CT scanner to be employed in-line with the device or installed with a new unit. The system will share the CT device’s couch, which will minimize the need for patient repositioning. The existing CT unit will not need to be modified, except for an extension of the CT patient table and possibly an increase in its range of motion. The CT table will need to be extended by an amount equal to the distance from CT isocenter to Linac isocenter; the patient will be initially positioned with the head near the superior edge of the table and the table near the inferior motion limit. In this position, the external patient markings will be aligned with the CT isocenter. Once the treatment plan is completed, the patient will be advanced by the distance between CT and Linac isocenter to prepare the patient for treatment. A manual movement of the patient from CT isocenter to Linac isocenter is planned. The CT room will possibly need to be extended to accommodate the increased footprint of the CT/SimpleXRT combined system. While modern CT tables are designed to support a 500 lbs patient, with the needed extension of the CT table, table weight allowance will need to be reduced accordingly.

The edges of the high-energy 6 MV treatment beam will have a minimum distance of 35 cm from any CT simulator radio-sensitive components such as the CT detectors or CT control electronics and receive no more than 3.0 Sv per year. This exposure level will ensure that the life expectancy of such CT components will not be shorter than the system life expectancy.

Shielding design

The radiation produced by the 6 MV Simple XRT poses a significant risk to radiation workers and members of the public in proximity. During treatment, this system will produce primary radiation from the treatment beam, which is transmitted through the patient. Additionally, it will generate secondary radiation due to scattering events from within the patient, and leakage radiation due to radiation escaping from the collimating and X-ray generating components. This radiation emanating from the Simple XRT needs to be minimized to decrease the risk posed to patients and workers.

The system utilizes passive methods to minimize radiation levels and shielding requirements. For typical vault design, a primary shielding belt intercepts the projected primary radiation field, by creating an attenuating barrier following the projection of the treatment field against the walls and ceiling. The Simple XRT system moves this primary shield close to the patient, thus reducing the shielded area dramatically. Compared to conventional linac systems with a source-to-axis distance of 1 m, the proposed system will shorten that distance to 0.85 m. This also causes an increase in the dose rate at the isocenter, thereby decreasing the typical treatment time and utilization factor. Due to the proposed system’s maximum field size of 25 cm x 25 cm, the width of the primary shield will be reduced compared to the typical linac field size. Furthermore, unlike systems such as the CyberKnife, the Simple XRT system is restricted to move along a circular path. This restriction alone will greatly reduce the area required for the primary shielding.

**Figure 2:**
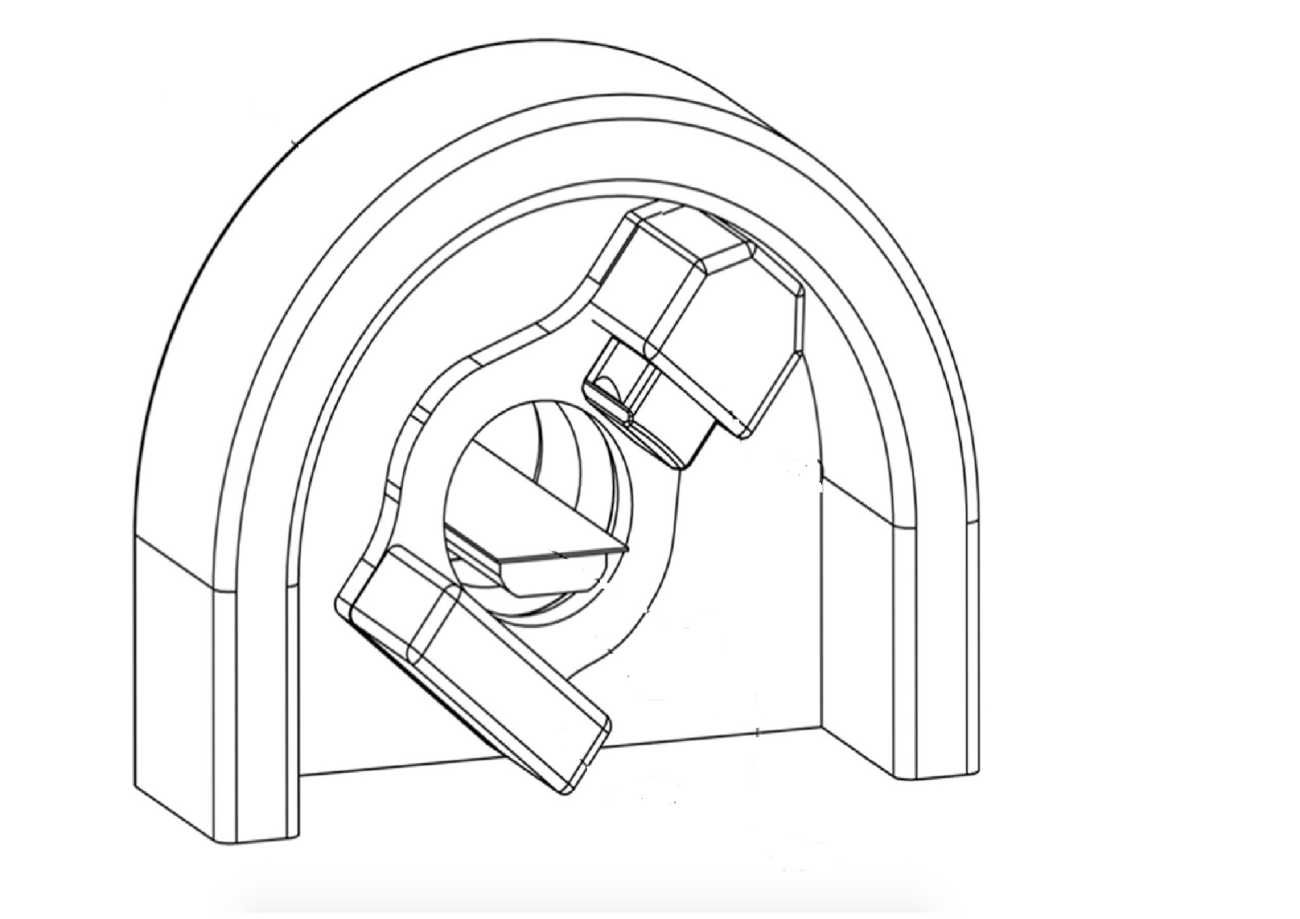
Proposed configuration of Simple XRT shows the primary shielding and beam stop Image courtesy ETM Electromatic Inc., Newark, CA.

To address the primary radiation, the Simple XRT system will attenuate the treatment beam radiation via a solid arch made of lead. This lead arch, shown in Figure 2, will intercept the treatment beam at every gantry angle. Additionally, a lead beam stopper will be mounted downstream of the isocenter opposite to the treatment head to further attenuate the primary radiation. The floor mounted arch along with the beam stopper combine to make up 5 tenth value layers (TVL) of lead for the 6 MV photon energies. A TVL is a layer of attenuating material that will reduce the incoming radiation intensity to one-tenth of the incoming value. According to National Council on Radiation Protection and Measurements report #151 (2005), a single TVL of lead has a thickness of 5.7 cm for 6 MV, therefore the combined thickness of the primary shield will be 28.5 cm. This arch will be floor mounted and is equidistant from the isocenter for all angles.

The scattered radiation is mainly attenuated by the lead arch, but there is some scattered radiation that will not be intercepted by the primary shield. The scattered radiation that is not intercepted will be absorbed by a 3-inch steel shield that can be mounted in the walls of the treatment room. Furthermore, there will be radiation emitted from the collimating head of the Simple XRT. This leakage radiation will be attenuated by the lead shielding mounted surrounding the treatment head of the system. That portion of the radiation that is not absorbed by the treatment head shielding will be absorbed by the lead arch.

To keep radiation risk to a minimum, shielding dose limits for the public of 1 mSv/y (100 mrem/y) and 5 mSv/y (500 mrem/y) for radiation workers will need to be observed. The final shielding design for the Simple XRT treatment room will need to take machine utilization, occupancy factors, and use factors into account. Assuming the surrounding rooms are always occupied during treatment, an occupancy factor of 1.0 is assigned; due to the rotational nature of the treatment delivery, an angular use factor of 0.1 will be employed. With a maximum field size of 25 cm x 25 cm, a rotational arc section of 16.4 deg will be occupied by that beam. As a complete arc is comprised of 270 deg, 16.5 projections of the maximum field size beam could be arranged along the arc without overlap. Therefore, a conservative use factor of 1/10 was applied as most treatments will be delivered as rotational arcs. Assuming that the system will only be delivering radiation 10% of the total time allotted for each patient, a utilization factor of 0.1 is assigned. The nearest patients or radiation workers will be assumed to be approximately 3.5 m away from the treatment isocenter. Due to the inverse square fall-off of the radiation intensity, this will introduce a correction factor of 0.081. With a dose rate of 300 MU/min is equivalent to 300 cGy/min or 300,000 mrem/min at isocenter. Due to the applied 5 TVL of primary shielding in the arch, the instantaneous dose rate is reduced to 3 mrem/min downstream of the lead arch. After applying the correction factors of 0.081 (inverse-square correction), 0.1 (utilization factor), and 0.1 (use factor), the expected dose rate will be 0.00243 mrem/min, or 291.6 mrem/year which is above the acceptable level of 100 mrem/year (1 mSv/year) for the public dose. Therefore, an additional 0.5 TVLs will need to be added to the primary shielding barrier in the wall.

**Figure 3:**
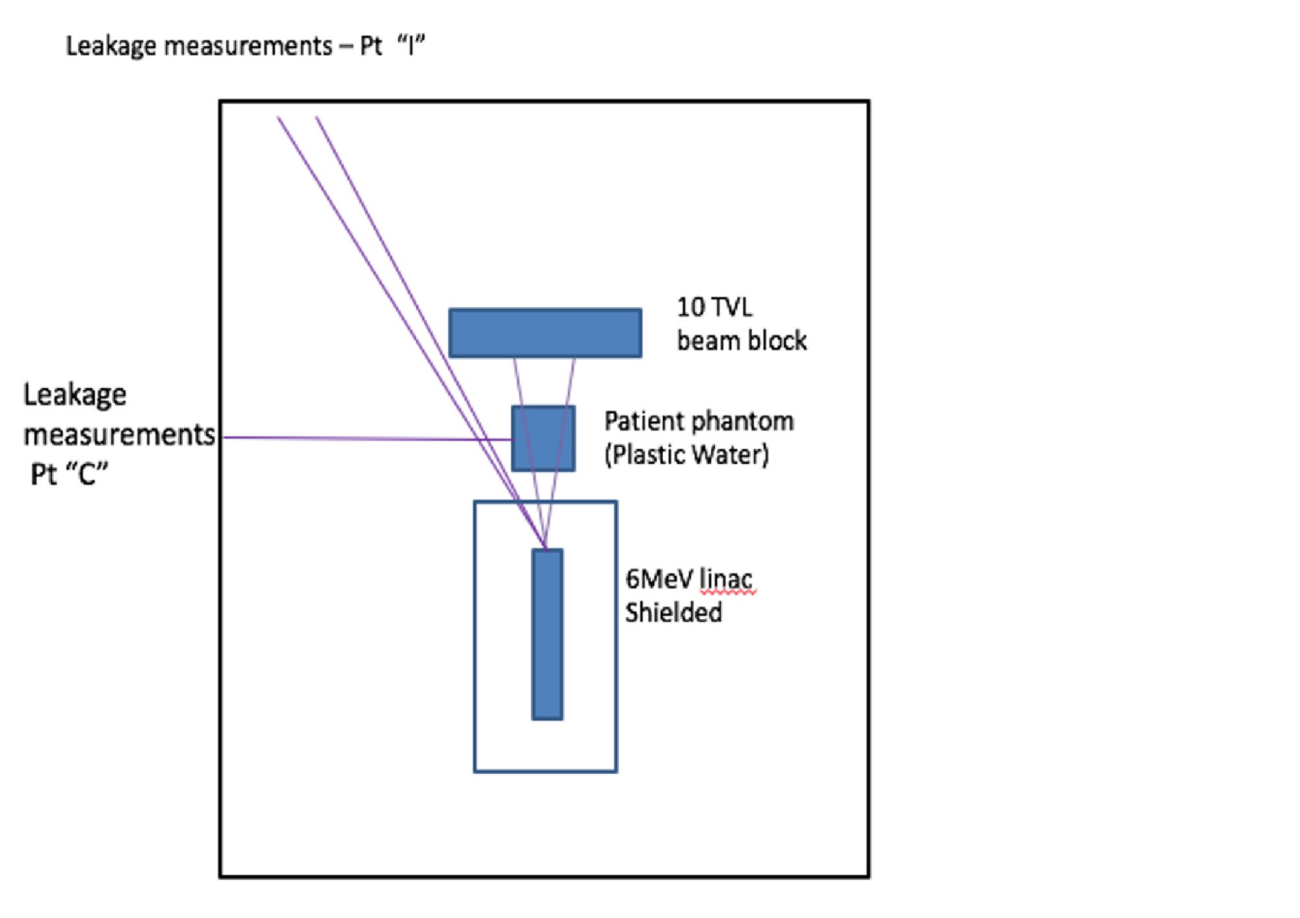
Radiation shielding experimental setup Pt: point; TVL: tenth value layer Image courtesy ETM Electromatic Inc., Newark, CA.

The feasibility of this theoretical calculation is confirmed by the following experiment, shown in Figure 3. The treatment parameters of the Simple XRT system were replicated in order to accurately quantify the radiation exposure at various points. These replicated features included an identical source to axis distance of 85 cm, source to beam stop distance and thickness, and steel shielding. The experiment used a 6 MV linac with the same dose rate as used in the proposed Simple XRT device. A solid water phantom was used to represent a patient. Radiation leakage measurements were made in two locations to determine the exposure rate. The experiment confirmed that the largest component of scattering radiation was due to scattering from the simulated patient phantom.

To quantify the exposure rates due to secondary radiation, two measurement locations were chosen to represent the differing intensities. At point “C”, a radiation scatter measurement was taken along the longitudinal axis at 2.8 m for the isocenter, which resulted in an exposure rate of 145.0 mR/hr. However, once shielding plates of 3” steel were added, this exposure rate drops to 3.6 mR/hr. At point “I”, a radiation scatter measurement was acquired at a location as close as possible to the central axis that was not blocked by the beam stopper at a distance of 3.66m from the isocenter. This position, which represents the largest secondary radiation intensity, produced an exposure rate of 330.0 mR/hr, but is reduced to 23.0 mR/hr with 3” of steel shielding added upstream. An expected annual exposure and dose at points “C” and “I” can be determined by applying the previously stated correction factors. With a use factor of 0.1, a utilization factor of 0.1, and an inverse square correction factor of 1.0, the resulting exposure rate at location “C” and “I” will be approximately 0.036 mR/hr and 0.23 mR/hr, respectively. Assuming 2000 hours in an annual work year, the calculated exposure and dose rates become 72.0 mR/y or 0.72 mSv/y and 460 mR/y or 4.6 mSv/y at points “C” and “I”, respectively. These calculated dose rates are less than the shielding annual limit for radiation workers of 5.0 mSv/y and less than 10% of the annual permissible dose of 50 mSv for occupationally exposed personnel. Therefore, the results of this experiment support the argument for minimal required shielding of the Simple XRT room. With the proposed parameters and shielding, a conventional treatment bunker is not necessary.

In comparison, a standard radiation bunker requires a primary radiation barrier of 6 to 7 feet of concrete for the primary shield and 3 to 4 feet for all other secondary shielding barriers. This will typically results in 700 to 1,000 tons of concrete with a cost of 1.5 to 2.5 million USD. The proposed system will require 3 to 4 inches of steel plating strategically placed at locations of highest radiation levels as described above at an estimated cost of 15,000 to 25,000 USD.

Device comparison

The proposed system’s treatment head will rotate on a circular gantry at a speed of 1 rotation per minute, or 6° per second, with a maximum dose rate of 300 cGy/min at 6 MV and in order to avoid interference with the treatment couch, the system will rotate through a 270 degrees arc. The MLC, will support a field size of 25 cm x 25 cm as mentioned earlier, and will provide a 0.5 cm leaf width at the isocenter. Although large field sizes are possible, it would require modifications to make the primary shielding arch wider.

The proposed Simple XRT system offers a wide array of benefits and possible applications within the field of radiotherapy. The proposed device is compared with current radiotherapy systems, offering similar features and capabilities, as shown in Table 1. The cost estimate of 1 million USD is based on a conceptual bill of materials (BOM) generated for this device. This cost does not include the cost of the CT scanner and cost for additionally needed electrical and HVAC system.

**Table 1:** Comparison of various radiation therapy devices MLC: multi-leaf collimator; TBI: total-body irradiation; IMRT: intensity modulated radiation therapy; 3D: three-dimensional; CRT: conformal radiation therapy; IGRT: image-guided radiotherapy; VMAT: volumetric modulated arc therapy; EPID: electronic portal imaging device; MVCT: megavoltage computed tomography; CBCT: cone‐beam computed tomography; MRI: magnetic resonance imaging; CT: computed tomography.

Criteria	Varian Unique	Accuray Tomotherapy	Accuray Cyberknife	Elekta Synergy	ViewRay MRIdian	BrainLab Vero	SimpleXRT
Photon Energies (MV)	6	6	6	6/10/15/18	1.17/ 1.33 MeV (now 6MV)	6	6
Isocentric/ NonIsocentric	Iso	Iso	Both	Iso	Iso	Both	Iso
Max Dose Rate (cGy/Min)	600	850	1000	600	500	500	600
Source Type	S-Band Linac	S-Band Linac	X-Band Linac	S-Band Linac	3 Co-60 sources or Linac	C-Band Linac	S-Band Linac
Field Definition	120 Leaf Millenium MLC	Dynamic 64 leaf MLC, X-ray Jaws	Iris Variable collimator 5-60 mm or InCise 41 leaf pair MLC	80 Leaf MLC or 160 leaf Agility MLC	Three 60 leaf MLCs, Double Focused	Gimbaled treatment head, 60 leaf MLC	MLC, X-ray Jaws
Leaf Width	0.5 cm	0.625 cm	0.25 cm	0.4 cm or 0.5 cm	1.05 cm	0.25 cm	0.5 cm or 0.3 cm
Max Field Size	40 cm x 40 cm	5 cm x 10 cm	5 mm-60 mm or 10 cm x 12 cm	40 cm x 40 cm	30 cm x 30 cm	15 cm x 15 cm	25 cm x 25 cm
SRS/SBRT	Both	Both	Both	Both	Both	Both	Both
Treatments	Rapid Arc	TBI, IMRT, 3D CRT	IGRT	VMAT, 3D CRT	3D CRT, IMRT	VMAT, IMRT, Hybrid Arc	IGRT, VMAT, IMRT
Image Guidance	EPID	MVCT	Stereoscopic kV X-ray	CBCT, EPID, Stereoscopic kV X-ray	Real Time MRI	Stereoscopic kV X-ray, Fluoroscopic, CBCT, EPID	kV Fan Beam CT
Tumor Tracking	-	-	Multiple tracking systems	Motion View, fluoroscopic imaging	Real Time MRI	Infared, predictive algorithms	-
Vault	Vault Required	Self-Shielded	Vault Required	Vault Required	Vault Required	Self-Shielded/Vault Req.	Minimal Shielding
Approximate System Cost (Million USD)	$2	$3.7	$7.2	$2	$5.2	$6.5	$1

## Discussion

The Simple-XRT can be compared to modern radiotherapy systems, due to its ability to provide VMAT, SRS/SBRT, and diagnostic quality imaging. The similar treatment ability coupled with its low cost, reduced facility shielding, and speed of installation makes this device applicable in numerous radiotherapy settings. The Simple-XRT device can provide benefits to already robust radiation therapy centers, rural areas, primary care hospitals, and areas that have little to no access to radiation therapy resources.

Typically, radiotherapy systems are utilized within a radiotherapy clinic, where patients are sent from the primary care institution to the clinic for outpatient daily treatment. This process may prove difficult for patients that have been hospitalized for other reasons. Transportation to and from these clinics can require ambulance rides with complex required medical equipment. This process can prove to be costly and hard on the patient, but a solution that is proposed with this system is to install an onsite treatment room at the primary care facility, to treat patients that may have a difficult time moving off-site. This solution will provide an option for patients in an immobile situation to receive the care that is needed at a relatively small investment to the primary care facility. Due to the inherent shielding effect of this system, the primary care facility can use virtually any room with sufficient size, and ability to support the additional steel shielding as the treatment room.

While the Simple XRT system can be used as the primary treatment system, many clinics can have multiple high-end systems that are a system of choice due to the added features and patient throughput. In this situation, the SimpleXRT system can be used as an overflow system to either absorb increased patient load, or to relieve the strain on the higher end systems. Since the proposed system has the ability to be implemented within weeks and can treat most indications of cancer, it can serve as a backup system whenever a large influx of patients causes an inundation within a clinic. As many indications of cancer can be treated without the high-end features, it may prove beneficial to shift some of the patient load to the Simple XRT in order to preserve the high-end systems for indications that require the superior precision and the additional features.

Future developments will include automation of some of the treatment planning steps, image-based verification of the shift from imaging isocenter to treatment isocenter, automation of the imaging process, and patient treatment delivery. Treatment planning can be partially automated by utilizing an algorithmic approach. Historic methods and common anatomy atlases will be used to simplify this process. With each treatment plan, the planning templates and anatomy atlases will be amended. Secure cloud sharing according to the Healthcare Information Patient Privacy Act (HIPPA) will add the ability to share patient data among hospitals and treatment centers. By sharing patient data and assembling a cancer registry, the entire community of users will benefit and improve patient treatments. Future application of artificial intelligence is considered to predict treatment parameter deviations from treatment standards, treatment outcomes, and complications.

## Conclusions

With the Simple XRT system's imaging technology and maximum field size, most radiotherapy indications can be treated. The proposed system will enable poor or rural areas that do not currently have feasible options for radiotherapy to invest in a low-cost system providing treatments. Given that the construction effort will be reduced, an estimate for installation was placed at four weeks, which is much quicker than the 1-1.5 years needed for many conventional systems. While this system can equip rural and poor areas with a cost-effective radiotherapy system, areas with a very sparse population density may benefit from a mobile radiotherapy site. Due to the small amount of shielding required, the proposed system can be modified to become mobile. This will allow the radiotherapy system to travel to under-served and rural areas that may not have a nearby radiation therapy clinic.

At a very low comparative cost, the Simple XRT system provides state-of-the-art fan beam CT IGRT and addresses the needs that are currently underrepresented in the current market of radiation therapy, which is the need for a low cost system that does not require expensive construction and infrastructure, can be installed relatively quickly, provide treatment for most cancer cases, and provides a solution to those areas and communities that are in need of radiotherapy treatment ability.
